# GDIv2: improving variant selection from human exomes

**DOI:** 10.1093/bioadv/vbag144

**Published:** 2026-05-23

**Authors:** Estelle Talouarn, Yoann Seeleuthner, Astrid Marchal, Clément Conil, Jean-Laurent Casanova, Peng Zhang, Laurent Abel, Yuval Itan, Aurélie Cobat

**Affiliations:** Laboratory of Human Genetics of Infectious Diseases, Necker Branch, INSERM U1163, Paris, France; Imagine Institute, Paris City University, Paris, France; Laboratory of Human Genetics of Infectious Diseases, Necker Branch, INSERM U1163, Paris, France; Imagine Institute, Paris City University, Paris, France; Laboratory of Human Genetics of Infectious Diseases, Necker Branch, INSERM U1163, Paris, France; Imagine Institute, Paris City University, Paris, France; Laboratory of Human Genetics of Infectious Diseases, Necker Branch, INSERM U1163, Paris, France; Imagine Institute, Paris City University, Paris, France; Laboratory of Human Genetics of Infectious Diseases, Necker Branch, INSERM U1163, Paris, France; Imagine Institute, Paris City University, Paris, France; St. Giles Laboratory of Human Genetics of Infectious Diseases, Rockefeller Branch, The Rockefeller University, New York, NY, United States; Pediatric Hematology-Immunology and Rheumatology Unit, Necker Hospital for Sick Children, Assistance Publique-Hôpitaux de Paris (AP-HP), Paris, France; Howard Hughes Medical Institute, New York, NY 10032, United States; Laboratory of Human Genetics of Infectious Diseases, Necker Branch, INSERM U1163, Paris, France; Imagine Institute, Paris City University, Paris, France; St. Giles Laboratory of Human Genetics of Infectious Diseases, Rockefeller Branch, The Rockefeller University, New York, NY, United States; Laboratory of Human Genetics of Infectious Diseases, Necker Branch, INSERM U1163, Paris, France; Imagine Institute, Paris City University, Paris, France; St. Giles Laboratory of Human Genetics of Infectious Diseases, Rockefeller Branch, The Rockefeller University, New York, NY, United States; The Windreich Department of Artificial Intelligence and Human Health, Icahn School of Medicine at Mount Sinai, New York, NY 10029, United States; Mindich Child Health and Development Institute, Icahn School of Medicine at Mount Sinai, New York, NY 10029, United States; Laboratory of Human Genetics of Infectious Diseases, Necker Branch, INSERM U1163, Paris, France; Imagine Institute, Paris City University, Paris, France; St. Giles Laboratory of Human Genetics of Infectious Diseases, Rockefeller Branch, The Rockefeller University, New York, NY, United States

## Abstract

**Motivation:**

The Gene Damage Index (GDI) quantifies the cumulative mutational damage of protein-coding genes in the general population and helps prioritize candidate disease genes in sequencing studies. However, the original GDI is influenced by coding sequence length and does not account for gene-specific differences in variant deleteriousness. We developed GDIv2, an updated framework correcting for coding sequence length and incorporating gene-specific normalization of CADD scores to improve discrimination between disease-relevant and non-relevant genes.

**Results:**

Four GDIv2 implementations were generated using 1000 Genomes Project and gnomAD datasets for both GRCh37 and GRCh38 genome builds. Benchmarking against the original GDI showed that all GDIv2 versions significantly improved discrimination between relevant and accessory genes, reduced erroneous exclusion of relevant genes, and increased exclusion of accessory genes. GDIv2_1kGP_37 achieved the best AUC performance and excluded 24.6% of accessory genes while retaining 96.7% of relevant genes. Compared with RVIS, LOEUF, s_het_, and CoNeS, GDIv2_1kGP_37 performed similarly in AUC analyses. Combining GDIv2_1kGP_37 with CoNeS and LOEUF further improved filtering, excluding 42.7% of accessory genes while removing only 2.4% of relevant genes.

**Availability and implementation:**

GDIv2 resources are freely available at https://hgidsoft.rockefeller.edu/GDI/GDIv2.html.

## 1 Introduction

Several gene-level metrics have been developed based on public databases of human exomes and genomes, for quantifying the degree of intolerance to mutation or gene constraint [e.g. pLI (Karczewski *et al.* 2020), LOEUF (Karczewski *et al.* 2020), RVIS (Petrovski *et al.* 2013) and its subRVIS extension (Gussow *et al.* 2016)], the mutational burden [e.g. GDI (Itan *et al.* 2015)], and the level of negative selection on each gene [e.g. s_het_ (Zeng *et al.* 2024), CoNeS (Rapaport *et al.* 2021)]. pLI and LOEUF quantify the difference between the observed number of loss-of-function (LoF) variants and the number expected under a model of no selective constraint. They have been broadly used to help identify genes in which a single disrupting mutation is likely to be clinically important (Fuller *et al.* 2019). In the RVIS method, the number of common variants (missense and pLoF) is regressed against the total number of variants in the gene. The regression residuals then constitute the residual variation intolerance score (RVIS), and subRVIS is an extension of this method in which genes are further divided into protein-coding domains or exons. The s_het_ framework combines a flexible population genetics model of LOF allele frequencies to obtain the likelihood of observing a LOF at a given frequency, combined with machine learning on thousands of gene features to improve estimates for genes with few expected LOFs and was shown to perform well on short genes. The CoNeS score was developed as a composite score for the detection of negative selection based on intra- (including LOEUF and pLI) and interspecies indicators. Not only do these scores improve our understanding of the functional importance of human genes, but they can greatly facilitate the interpretation of individual patient sequence data.

In this context, we previously developed the Gene Damage Index (GDI) (Itan *et al.* 2015), a gene-level metric that integrates both the allele frequency spectrum with the deleteriousness predictions from CADD (Rentzsch *et al.* 2019) for predicted LOF, missense and inframe indel variants, to identify genes with a high level of mutational damage in the general population. We showed that genes accumulating LOF, missense and inframe indels with high CADD score in the general population are unlikely to cause life-threatening diseases but make a disproportionate contribution to the variant calls observed in any given patient. Conversely, mutations in genes that are never, or only rarely mutated under normal circumstances are more likely to underlie life-threatening conditions. The GDI efficiently filters out variants unlikely to underlie life-threatening conditions from patient sequencing data and has been successfully used in multiple studies (Hernandez *et al.* 2018, 2019, Yang *et al.* 2020, Lee *et al.* 2023). However, it did not account for the length of the coding sequence, with which it is strongly correlated (Itan *et al.* 2015). Consequently, the TTN gene, which encodes the largest known protein, had the highest GDI value, although mutations in this gene can cause autosomal recessive (AR) and autosomal dominant (AD) cardiomyopathy (Herman *et al.* 2012, Fomin *et al.* 2021). Subsequently, we demonstrated that the distributions of CADD scores for disease-associated mutations curated in the HGMD database (Stenson *et al.* 2020) are gene-specific, with minimal overlap among the 95% confidence intervals across genes (Itan *et al.* 2016). We therefore proposed the gene-specific mutation significance cutoff (MSC), which provides low/high phenotypic impact cutoff values to improve the use of existing variant-level methods (Itan *et al.* 2016). Here, we propose a new formulation of the GDI (GDIv2) to take into account more effectively (i) the length of the coding sequence and (ii) the gene-specific CADD score distribution. We computed GDIv2 scores for each protein-coding gene, from two datasets (1kGP or GnomAD) and for two genome builds (GRCh37 and GRCh38). We compared GDI and GDIv2 performance to four gene-based scores developed for different purposes, namely RVIS, LOEUF estimated from GnomADv4.1, s_het_ and CoNeS.

## 2 Methods

### 2.1 Original GDI formulation

The GDI is designed to estimate the cumulative mutational damage to each human protein-coding gene in the general population. In the original version, the GDI raw score was calculated, by gene, as the sum of minor allele frequencies for all non-synonymous variants of the gene in the general population, weighted by their predicted damage score. GDI made use of the 1000 Genomes Project (1kGP) Phase 3 genetic variants call set, which was generated based on a combination of low-coverage WGS (mean depth 7.4×), high-coverage whole-exome sequencing (WES, mean depth 65.7×), and microarray genotyping data for lymphoblastoid cell line (LCL) samples derived from 2504 healthy individuals from 26 populations (Byrska-Bishop *et al.* 2022). The functional consequences of the variants for the genes were predicted with ANNOVAR (Wang *et al.* 2010) using the default parameters and transcript dataset and keeping the worst annotation across transcripts. The predicted damage score used to weight the variants (missense/nonsense/frameshift/in-frame indels/splice) was the CADDv1.3 score (Kircher *et al.* 2014). Since population frequency information is incorporated during CADD training, using raw CADD scores introduces a systematic bias when aggregating variants at the gene level. In the original GDI formulation, this effect was mitigated by standardizing CADD scores through division by the median CADD score of all non-synonymous variants within the same allele-frequency bin at the genome-wide level, a procedure that was shown to significantly improve GDI performance.

### 2.2 GDIv2 formulation

In GDIv2, we propose two key improvements: (i) we regress the raw GDI score against the length of the coding sequence to correct for gene-size effects. For a given transcript, the raw GDI score is computed as:


$${\mathrm{GDI}}_{\mathrm{raw}}=\sum_{i=1}^{n}{\mathrm{CADD}}_{i}^{\mathrm{std}}\times {\mathrm{freq}}_{i}$$

where *n* is the number of non-synonymous variants in the transcript, ${\mathrm{freq}}_{i}$ is the allele frequency of variant i, and ${\mathrm{CADD}}_{i}^{\mathrm{std}}\;$denotes the standardized CADD score of variant i. A linear regression model is then fitted:


$${\mathrm{GDI}}_{\mathrm{raw}}∼\mathrm{coding}\;\mathrm{sequence}\;\mathrm{len}gth.$$

The residuals of this regression are ranked across all genes and expressed as a PHRED score:


$$GDI_{\mathrm{Phred}}=-10\times \log10(\frac{\mathrm{rank}}{\mathrm{number}\;of\;\mathrm{genes}})$$

This transformation yields higher scores for genes that accumulate an unexpectedly high mutational burden relative to their coding sequence length, and lower scores for long genes with few and/or rare variants; (ii) Standardization of the CADD score at the gene-level. Indeed, we previously showed that the CADD score pattern of reportedly disease-causing variants varied greatly between genes (Itan *et al.* 2016). We therefore propose to standardize the CADD score of each variant by the median CADD score for all non-synonymous variants from the same minor allele frequency (MAF) bin [(≤10–4), (10–4—10–3), (10–3—10–2), (10–2—10–1), (10–1—0.5)] at the gene level (as opposed to genome-wide in the original formulation).

### 2.3 GDIv2 data sources

GDIv2 was computed using four large-scale population datasets: (i) the initial 1kGP Phase 3 genetic dataset aligned to the GRCh37 reference genome (GDIv2_1kGP_37); (ii) the 1kGP Phase 4 high-coverage WGS dataset, derived from an expanded cohort of >3200 individuals and aligned to GRCh38 (Hanchard and Choudhury 2022) (GDIv2_1kGP_38); (iii) the gnomADv2.1.1 (Karczewski *et al.* 2020) dataset, comprising 125 748 WES and 15 708 WGS aligned to GRCh37 (GDIv2_GnomAD_37); and (iv) the gnomADv4.1(Chen *et al.* 2024) dataset, comprising 730 947 WES and 76 215 WGS aligned to GRCh38 (GDIv2_GnomAD_38).

Other updates from GDI to GDIv2 included the use of CADD v1.7 (Schubach *et al.* 2024) for variant deleteriousness scoring and Ensembl VEP (McLaren *et al.* 2016) for functional annotation. For GDIv2_1kGP_38 and GDIv2_GnomAD_38, a single functional consequence per overlapping gene was retained for each variant, prioritizing the MANE transcript when available and otherwise the Ensembl canonical transcript. For GDIv2_1kGP_37 and GDIv2_GnomAD_37, a single functional consequence per overlapping gene was retained for each variant based on the Ensembl canonical transcript. GDIv2 calculations were restricted to 19 548 protein-coding genes with a MANE or a canonical transcript from the Gencode v39 transcript set. For GRCh37-based datasets, gene identifiers were matched to their Gencode v19 counterparts using ENSG identifiers and gene names, yielding 18 919 genes, of which 18 868 were associated with the same transcript in both GRCh37 and GRCh38.

### 2.4 Other gene-level metrics

RVIS and CoNeS were obtained from the study by Rapaport *et al.* (2021) and matched to GDI by gene identifiers. LOEUF scores were retrieved from GnomAD v4.1.1 (lof.oe_ci.upper) and matched to GDI using transcript identifiers. Selective constraint against heterozygous loss-of-function variants (s_het_) was derived from the posterior mean of the GeneBayes model, as described in the original publication (Zeng *et al.* 2024) and matched to GDI by gene identifiers.

### 2.5 Gene categories

We defined three gene categories for further performance assessment: relevant genes (N = 3076), accessory genes (N = 255) and background genes (N = 12 966). The relevant gene list was defined as the union of genes curated in the Gene-Disease Validity classifications (accessed on 2026/04/06) from the Clinical Genome Resource (www.clinicalgenome.org) (Rehm *et al.* 2015) with a Definitive or Strong level of evidence, as well as a set of inborn errors of immunity genes that are well established (Poli *et al.* 2025). This resulted in a total of 2197 genes. Mode of inheritance information was available for all genes and is provided in Table S2. The accessory gene set was defined as the union of three categories: (i) Olfactory receptor (OR) genes. We included all genes from the HGNC olfactory receptor gene group (https://www.genenames.org/data/genegroup/#!/group/141), which comprises 873 genes, including 419 annotated with a protein product and 390 classified as protein-coding in GENCODE v39. These genes are well known to be highly redundant and tolerant to variation; (ii) Testis-specific protein-coding genes. We added genes with highly restricted expression in testis, which are unlikely to be involved in severe systemic disease. These were defined using GTEx v11 (GTEx_Analysis_2025-08-22_v11) with the following criteria: TPM in testis > 5 and maximum TPM across all other tissues < 0.5. This yielded 374 genes, of which 371 are not part of the OR gene group; (iii) Genes frequently deleted in the general population. We included genes with evidence of tolerance to large structural variation. Using gnomAD SV v4.1, we selected deletions passing all quality filters (PASS) and retained deletions overlapping >50% of the gene sequence. For each gene, we computed a cumulative minor allele frequency (cMAF) defined as the sum of allele frequencies of all such deletions. Genes with cMAF > 10% were classified as accessory. This resulted in 141 genes, of which 129 do not overlap with the OR or testis-specific sets (Fig. S1). Four genes assigned to the relevant gene category were also identified as having a high structural variant burden (SV cMAF > 10%). In addition, one gene from the relevant category met the criteria for testis-specific expression. These five genes were retained in the relevant gene category. The final accessory gene set corresponds to the union of the three categories, excluding 5 relevant genes, resulting in a total of 897 genes. Genes not included in any of the above categories were referred to as background genes.

### 2.6 Performance assessment

We first compared the distributions of GDI and GDIv2 scores within each gene category using the non-parametric paired Friedman test. We further evaluated the performance of each score by determining its ability to discriminate between relevant and non-relevant genes, between accessory and non-accessory genes and between relevant and accessory genes, for which all gene-level scores were available, using ROC curves and AUC. AUC were compared using the bootstrap method as implemented in the pROC package. Given the strong imbalance between gene categories, we complemented the ROC-AUC analyses by precision-recall (PR) analyses. For each pairwise comparison between gene categories, PR-AUC values were computed using the PRROC R package. For the comparison of GDI and GDIv2 with CoNeS, LOEUF estimated from GnomADv4.1, s_het_ and RVIS, we focused on a subset of 14 671 autosomal genes for which all gene-level scores were available. We also compared the proportion of genes removed for each category by each score when defining a cutoff capturing the 5% most ‘damaged’ genes for each scores (Table S1), using McNemar’s test for paired proportions and adjusting *P*value for multiple testing using the Benjamini–Hochberg procedure. Analyses stratified by mode of inheritance were restricted to the three most frequent categories: autosomal dominant (AD), autosomal recessive (AR), and X-linked (XL).

## 3 Results

### 3.1 Distribution of GDIv2 and comparison to GDI by gene category

**Figure 1:**
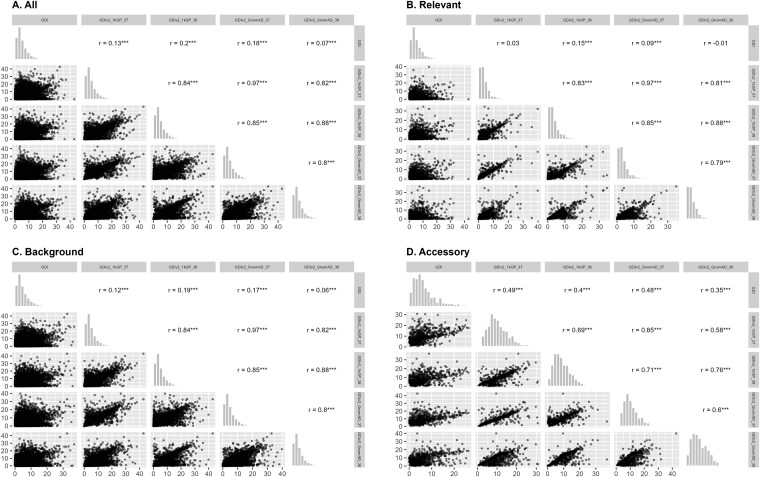
Pairwise correlations between GDI and GDIv2 for all genes (A), relevant genes (B), background genes (C) and accessory genes (D). r indicates the Spearman correlation coefficient. Number of * denotes statistical significance (*P < 0.05; **P < 0.01; ***P < 0.001).

We first examined the pairwise Spearman correlation between the GDI and the 4 versions of GDIv2 (Fig. 1). While there was a strong correlation between the four GDIv2 versions (Spearman r from 0.80 to 0.97), they were not or very weakly correlated to GDI (Spearman r from 0.07 to 0.18). This pattern was especially pronounced for relevant genes with Spearman correlation between GDIv2 versions and GDI ranging from −0.01 to 0.15 (Fig. 1B). GDIv2 scores correlated more strongly within the same genome build than within the same dataset, despite the much larger increase in sample size from 1kGP to GnomAD than from 1kGP phase3 to phase 4 or from GnomAD v2.1 to GnomAD v4.1 (Fig. 1). We further looked at the distributions of GDI and GDIv2 for several sets of genes (Fig. S2). As expected, for all five scores, the distributions differed significantly across the three gene categories, with accessory genes showing a shift toward higher values compared to relevant and background genes (Table 1 and Fig. S2). For the three gene categories, the distribution of the five scores significantly differed (PFriedman < 2 × 10^–16^). For accessory genes, the highest median was observed for GDIv2_1kGP_37 (median [IQR] = 8.77 [6.9]) and the lowest median for GDI (median [IQR] = 4.95 [5.93], *P*_GDI-vs-GDIv2_1kGP_37_ < 2 × 10^−16^). For relevant genes, the lowest median was observed for GDIv2_1kGP_38 (median [IQR] = 1.97 [3.65]) and the highest for GDI (median [IQR] = 3.31 [4.91], *P*_GDI-vs-GDIv2_1kGP_38_ < 2 × 10^−16^). Finally, for background genes, the highest median was observed for GDI (median [IQR] = 2.95 [4.62]) and the lowest for GDIv2_1kGP_38 (median [IQR] = 2.83 [4.46], *P*_GDI-vs-GDIv2_1kGP_38_ = 0.04).

**Table 1:** Median and interquartile range (IQR) of GDI and GDIv2 versions across gene categories.

	Accessory (n = 729)	**Background (n = 15** **281)**	Relevant (n = 2159)	*P* ^a^
**GDI**	4.95 (5.93)	2.95 (4.62)	3.31 (4.91)	< 2 × 10^−16^
**GDIv2 1kGP_37**	8.77 (6.9)	2.89 (4.48)	2.02 (3.54)	< 2 × 10^−16^
**GDIv2 1kGP_38**	7.26 (7.23)	2.83 (4.46)	1.97 (3.65)	< 2 × 10^−16^
**GDIv2 GnomAD_37**	8.19 (6.92)	2.89 (4.53)	2.09 (3.61)	< 2 × 10^−16^
**GDIv2 GnomAD_38**	7.44 (8.14)	2.87 (4.41)	1.91 (3.53)	< 2 × 10^−16^

a Kruskal-Wallis *P* from the comparison of the three gene categories.

**Figure 2:**
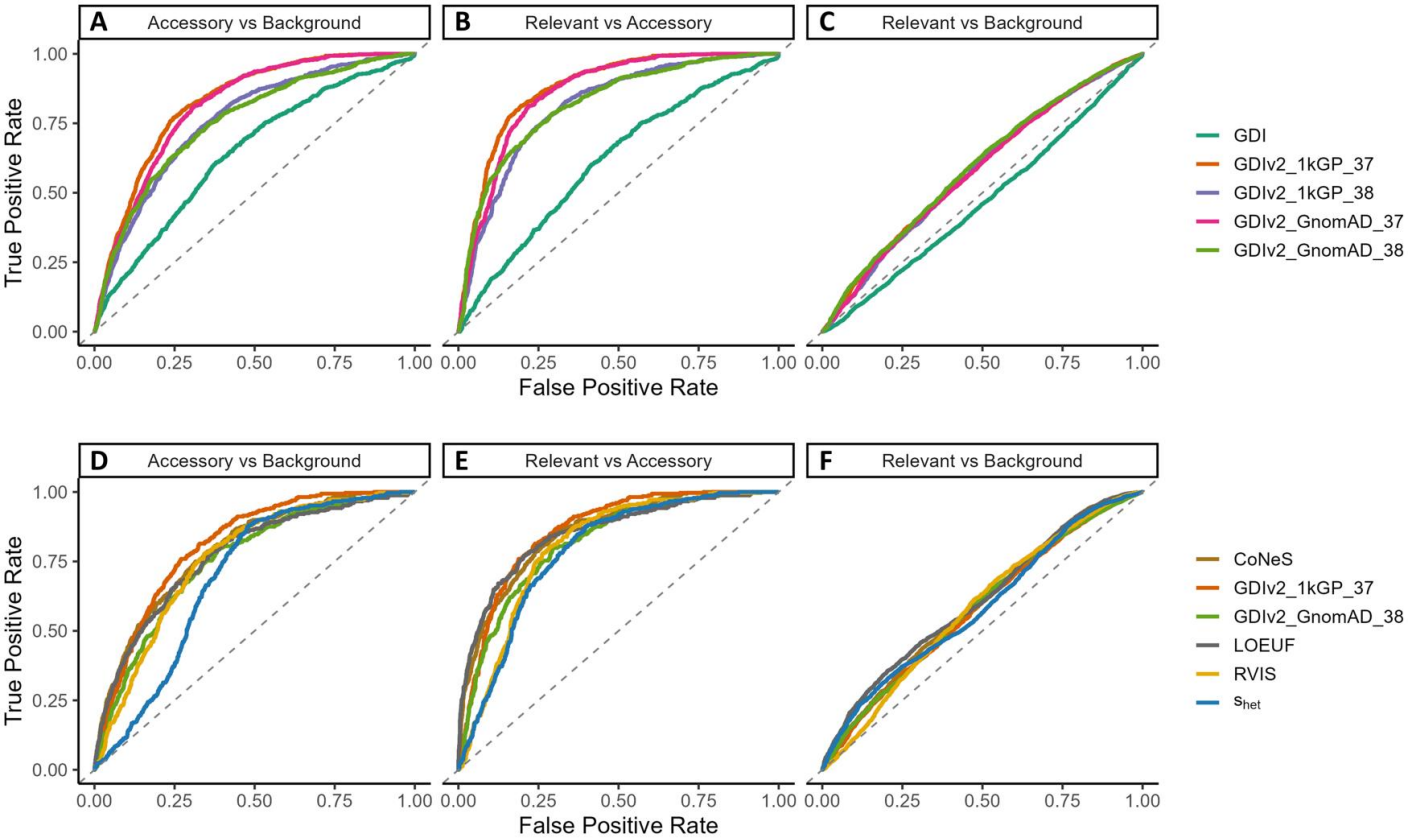
ROC curves assessing the ability of GDI and the four GDIv2 versions to separate relevant vs. background genes (A), relevant vs. accessory genes (B), and accessory vs. background genes (C); and of GDIv2_1kGP_37, GDIv2_GnomAD_38, RVIS, CoNeS, LOEUF_gnomADv4.1 and shet to separate relevant vs. background genes (D), relevant vs. accessory genes (E), and accessory vs. background genes (F).

Consistently, all GDIv2 versions performed significantly better than GDI in distinguishing relevant from accessory genes, with an AUC of 0.61 for GDI compared with 0.81 for GDIv2_1kGP_38, 0.82 for GDIv2_GnomAD_38, 0.86 for GDIv2_GnomAD_37 and 0.88 for GDIv2_1kGP_37 (all *P* < 10^−16^) (Fig. 2A, Table S3). The performance gain over GDI was mainly attributable to improved discrimination between accessory and background genes, although a significant enhancement was also observed for relevant versus background genes. GDIv2 versions based on the GRCh37 genome build showed a modest but significant improvement over those based on GRCh38 across all comparisons. A modest yet statistically significant advantage of the 1kGP-based GDIv2 was observed for GRCh37, whereas for GRCh38 the trend was reversed, with the gnomAD-based version showing superior performance (Table S3, Fig. 2B and C). Precision-recall AUC (PR-AUC) analyses were consistent with ROC-AUC results, with GDIv2_1kGP_37 and GDIv2_GnomAD_37 showing improved performance relative to their GRCh38 counterparts for distinguishing relevant and accessory genes (Table S4). Ablation analysis showed that regressing the raw score on coding sequence length yielded the largest improvement in the ROC-AUC performance for distinguishing relevant from accessory genes when comparing GDIv2_1kGP_37 with GDI (Fig. S3, Table S5).

### 3.2 Proportion of highly damaged genes by category

We further binarized the GDI and GDIv2 scores, using a threshold of 13 to separate the top 5% most damaged genes from the rest (Table 2). We reasoned that these genes (those lying above the threshold) would be unlikely to carry variants with strong phenotypic effects and could therefore be discarded from downstream analyses when searching for single-gene defects. Consistent with the quantitative and ROC-based analyses, GDIv2 significantly outperformed GDI. Specifically, GDI excluded a significantly higher proportion of relevant genes (6.7%) compared with GDIv2_scores (from 2.5% for GDIv2_GnomAD_38 to 3.7% for GDIv2_1kGP_38; all *P* < 10^–5^) and a lower proportion of accessory genes (12.9% for GDI versus up to 24.6% for GDIv2_1kGP_37; *P* = 2.7 × 10^−10^) (Table 2 and Table S6). Among GDIv2 implementations, GDIv2_1kGP_38 showed the least favorable filtering profile, excluding the lowest proportion of accessory genes (18.5%) and the highest proportion of relevant genes (3.7%), although differences in relevant gene exclusion were not statistically significant (Table S6).

**Table 2:** Number (proportion) of genes belonging to the top 5% of the distribution of GDI and GDIv2 scores.

Score	Accessory (N = 729)	**Background (N = 15** **281)**	Relevant (N = 2159)
**GDI**	94 (12.9%)	675 (4.4 %)	144 (6.7 %)
**GDIv2 1kGP_37**	179 (24.6 %)	681 (4.5 %)	71 (3.3 %)
**GDIv2 GnomAD_37**	164 (22.5 %)	687 (4.5 %)	76 (3.5 %)
**GDIv2 1kGP_38**	135 (18.5 %)	678 (4.4 %)	79 (3.7 %)
**GDIv2 GnomAD_38**	157 (21.5 %)	663 (4.3 %)	54 (2.5 %)

We further refined the analysis for relevant genes by stratifying them according to mode of inheritance (Table 3). A consistent pattern was observed for both autosomal recessive (AR) and autosomal dominant (AD) genes, with the original GDI excluding a higher proportion of relevant genes compared to all GDIv2 implementations. Among GDIv2 scores, GDIv2_1kGP_38 tended to exclude a slightly higher proportion of AR and AD genes than the other versions, whereas GDIv2_GnomAD_38 consistently excluded the lowest proportion. For X-linked (XL) genes, all methods excluded one gene (0.7%), although the specific gene differed across implementations. GDI excluded DMD, likely due to its large gene size, whereas GRCh37-based GDIv2 scores excluded OPN1LW, and GRCh38-based GDIv2 scores excluded RPGR. The different XL genes excluded by GRCh37- and GRCh38-based GDIv2 scores likely reflect build-specific effects rather than a robust biological difference. OPN1LW belongs to the OPN1LW/OPN1MW gene cluster, mutation of which underlie Blue cone monochromacy. This cluster consists of a single OPN1LW gene followed by one or multiple OPN1MW gene copies arranged in a tandem repeat structure (Wissinger *et al.* 2022). The sequences of OPN1LW and OPN1MW are highly homologous (>98% identity), including intronic and intergenic regions, which makes accurate read mapping challenging and limits the reliability of short-read sequencing approaches in this locus. RPGR is the main gene involved in X-linked retinitis pigmentosa. It contains 19 exons and has a complex transcript architecture, encoding around twenty transcripts (Fabard *et al.* 2026). Notably, the GRCh37- and GRCh38-based GDIv2 implementations were derived from different transcripts, with the GRCh38 transcript being shorter than its GRCh37 counterpart, which may contribute to the observed differences (Table S2).

**Table 3:** Number (proportion) of relevant genes belonging to the top 5% of the distribution of GDI and GDIv2 scores by mode of inheritance.

Score	AR N = 1278)	AD (N = 648)	XL (N = 144)
**GDI**	108 (8.5 %)	30 (4.6 %)	1 (0.7 %)
**GDIv2 1kGP_37**	57 (4.5 %)	8 (1.2 %)	1 (0.7 %)
**GDIv2 GnomAD_37**	58 (4.5 %)	12 (1.9 %)	1 (0.7 %)
**GDIv2 1kGP_38**	61 (4.8 %)	12 (1.9 %)	1 (0.7 %)
**GDIv2 GnomAD_38**	42 (3.3 %)	8 (1.2%)	1 (0.7 %)

### 3.3 Comparison with other gene-level metrics

**Figure 3:**
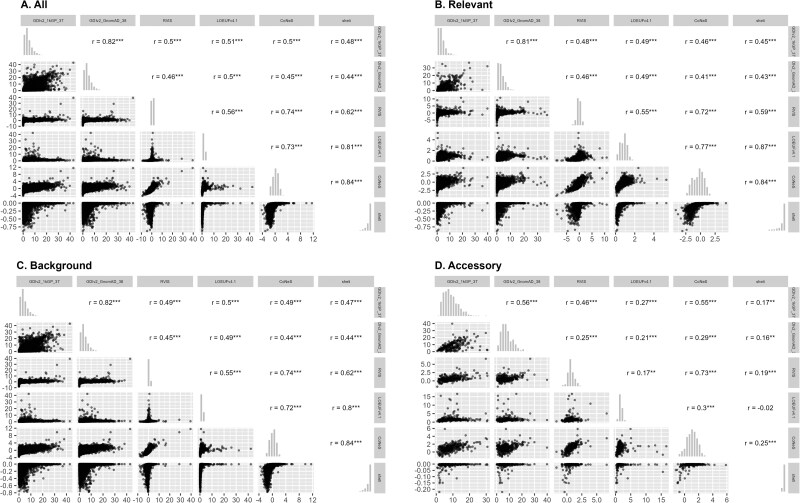
Pairwise correlations between GDIv2_1kGP_37, GDIv2_GnomAD_38, RVIS, CoNeS, shet and LOEUF for all genes (A), relevant genes (B), background genes (C) and accessory genes (D). r indicates the Spearman correlation coefficient. Number of * denotes statistical significance (*P < 0.05; ** P < 0.01; *** P < 0.001).

We then compared GDIv2_1kGP_37, which performed best in AUC-based analyses and excluded the largest proportion of accessory genes when binarized, and GDIv2_GnomAD_38, which excluded the lowest proportion of relevant genes, with other gene-level metrics, namely RVIS, LOEUF, CoNeS and s_het_. The distribution of RVIS, LOEUF, CoNeS and s_het_ across the three gene categories are shown in Fig. S4 and detailed in Table S7. Both GDIv2 scores showed moderate correlation with RVIS (Spearman’s r = 0.45 and 0.41 with GDIv2_1kGP_37 and GDIv2_GnomAD_38, respectively), LOEUF (Spearman’s r = 0.23 and 0.24), CoNeS (Spearman’s r = 0.51 and 0.47), and s_het_ (Spearman’s r = −0.25 and −0.24) (Fig. 3A). When assessing the ability to distinguish relevant from accessory genes, GDIv2_1kGP_37, CoNeS and LOEUF performed similarly with AUC of 0.86, 0.85 and 0.85, respectively, and significantly outperformed the other scores (Table 4, Table S8, Fig. 2E). In PR-AUC analyses, all scores performed well to distinguish between relevant and accessory genes (PR-AUC = 0.96–0.98), with GDIv2_1kGP_37 showing the highest PR-AUC, followed closely by CoNeS and LOEUF. When the classification was reversed to identify accessory genes against relevant genes, performance was lower overall, but LOEUFv4.1 showed the best discrimination, followed by CoNeS and GDIv2_1kGP_37 (Table S9).

**Table 4:** Area under the curve (AUC) values for discrimination between accessory, relevant, and background gene sets across scores.

	Accessory vs background	Relevant vs accessory	Relevant vs background
**GDIv2_1kGP_37**	0.81	0.859	0.575
**GDIv2_GnomAD_38**	0.753	0.815	0.583
**RVIS**	0.759	0.804	0.581
**LOEUF**	0.77	0.85	0.609
**CoNeS**	0.791	0.852	0.593
**s_het_**	0.693	0.786	0.584

We further compared the proportion of genes falling within the top 5% of each score distribution across the three gene categories (Table 5, Table S10). LOEUF excluded the smallest proportion of relevant genes (0.9%), followed by CoNeS (1.5%), s_het_ (2.2%), GDIv2_GnomAD_38 (2.3%), and GDIv2_1kGP_37 (3.0%), whereas RVIS showed the highest exclusion rate (4.7%). When stratifying relevant genes by mode of inheritance, RVIS excluded the highest number of XL genes (n = 4). CoNeS excluded the smallest proportion of AD genes (n = 4, 0.6%), while LOEUF excluded the smallest proportion of AR genes (n = 11, 0.9%). In contrast, CoNeS excluded the largest proportion of accessory genes (n = 84, 25.6%), substantially outperforming LOEUF (n = 74, 22.6%), GDIv2_1kGP_37 (n = 63, 19.2%), GDIv2_GnomAD_38 (n = 44, 13.4%), RVIS (n = 48, 14.6%), and s_het_ (n = 24, 7.3%). When binarized, these results identify CoNeS and LOEUF as the gene-level metrics achieving the most favorable balance between minimizing the exclusion of relevant genes and maximizing the exclusion of accessory genes.

**Table 5:** Number (proportion) of genes belonging to the top 5% of the distribution of the best performing GDIv2 scores, LOEUF, RVIS, s_het_, and CoNeS.

Score	Accessory (N = 328)^a^	**Background (N = 14** **042)^a^**	Relevant (N = 2104)^a^
**GDIv2_1kGP_37**	63 (19.2%)	557 (4.0%)	64 (3.0%)
**GDIv2_GnomAD_38**	44 (13.4%)	540 (3.8%)	48 (2.3%)
**RVIS**	48 (14.6%)	646 (4.6%)	99 (4.7%)
**LOEUF**	74 (22.6%)	519 (3.7%)	19 (0.9%)
**CoNeS**	84 (25.6%)	589 (4.2%)	31 (1.5%)
**s_het_**	24 (7.3%)	807 (5.7%)	46 (2.2%)
**Combined**	140 (42.7%)	1060 (7.5%)	51 (2.4%)

a Number of genes with complete data available for all scores.

### 3.4 Combining gene-level metrics to maximize filtering performance

CoNeS and LOEUF outperformed GDIv2_1kGP_37 when binarized but showed similar performance in terms of AUC. The modest correlation between the three scores suggests that they could provide complementary information. When combined, 47% of accessory genes fell in the top 5% of either the CoNeS, GDIv2_1kGP_37, or LOEUF distributions, compared with 6.1% of relevant genes. Among the 129 relevant excluded genes, 44 (34.1%) had a negative CoNeS score suggesting some level of negative selection, compared to 3 (1.9%) of the excluded accessory genes. Moreover, 72 excluded relevant genes (55.8%) had a LOEUF value < 1 compared to 11 (7.1%) excluded accessory genes. We further proposed to optimize our filtering strategy by rescuing genes in the top 5% of either the CoNeS, or GDIv2_1kGP_37, or LOEUF distributions with LOEUF score < 1 or CoNeS <0. With this strategy, we rescued 14 (9.1%) accessory genes and 78 (60.5%) relevant genes initially excluded. Overall, this led to the exclusion of 42.7% of accessory genes and only 2.4% of relevant genes, among which 0 were known XL, 40 were known AR genes and 9 were known AD (Table 6). To assess the robustness of this approach, we evaluated the filtering and rescue strategy using 5-fold cross-validation. This analysis showed consistent performance across folds, with a mean exclusion of accessory genes of 42.7% (from 38.5% to 45.4%) and a mean exclusion of relevant genes of 2.42% (from 1.4% to 3.1%), confirming a stable performance (Table S11). In total, 1251 genes (7.6%) were excluded by our filtering strategy and accounted for 7.7 to 15.2% of the total allele counts of candidate coding variants in the 1kGP datasets (Table S12).

**Table 6:** Number (proportion) of releavant genes belonging to the top 5% of the distribution of the best performing GDIv2 scores, LOEUF, RVIS, s_het_, and CoNeS by mode of inheritance.

Score	AR (N = 1250)^a^	AD (N = 630)^a^	XL (N = 137)^a^
**GDIv2_1kGP_37**	52 (4.2%)	6 (1.0%)	1 (0.7%)
**GDIv2_GnomAD_38**	38 (3.0%)	6 (1.0%)	1 (0.7%)
**RVIS**	78 (6.2%)	11 (1.7%)	4 (2.9%)
**LOEUF**	11 (0.9%)	7 (1.1%)	0 (0%)
**CoNeS**	24 (1.9%)	4 (0.6%)	0 (0%)
**s_het_**	34 (2.7%)	11 (1.7%)	0 (0%)
**Combined**	40 (3.2%)	9 (1.4%)	0 (0%)

a Number of genes with complete data available for all scores.

## 4 Discussion

We propose here an updated and improved formulation of the GDI to more accurately quantify the mutational burden of protein-coding genes in the human population. By incorporating regression on coding sequence length and gene-specific normalization of CADD scores, GDIv2 reduces biases driven by gene length and global deleteriousness calibration. Using multiple large-scale population datasets and genome builds, we showed that GDIv2 displays robust internal consistency. Despite the substantial increase in sample size from 1kGP to GnomAD, GDIv2 scores showed strong correlations within the same genome build for the two datasets. This suggests that the dependency of GDIv2 on sample size is limited, although the genome remains far from saturation at the vast majority of non-CpGs sites at current sample sizes (Agarwal and Przeworski 2021). In contrast, pLI and LOEUF, which rely on the presence or absence of variants without accounting for their frequency, are likely more sensitive to sample size. Because CoNeS incorporates both LOEUF and pLI in its calculation, some degree of sample-size dependency is expected, with potential improvement of CoNeS as LOEUF and pLI are computed from larger datasets.

Across all benchmarking analyses, GDIv2 outperformed GDI in discriminating relevant genes from accessory genes, excluding fewer disease-relevant genes and a larger fraction of accessory genes, and improving its utility as a first-pass gene-filtering tool in WES and WGS analyses. The ablation study indicated that accounting for coding sequence length was the primary contributor to the improvement from GDI to GDIv2. *TTN*, which plays a crucial role in cardiomyopathies and muscle disorders (Fomin *et al.* 2021, Vatta *et al.* 2025), provides a canonical illustration of this improvement. *TTN* ranked as the most damaged gene with GDI due to its exceptional length, whereas all versions of GDIv2 classify *TTN* as substantially less damaged, consistent with its relatively low LOEUF and intermediate CoNeS values. At the opposite end of the spectrum, pLI and LOEUF are known to exhibit limited power to identify constrained genes with short protein-coding sequence (Fuller *et al.* 2019, Zeng *et al.* 2024). Recently, the s_het_ framework was shown to improve constraint estimation for short genes with few expected LoFs by integrating several gene features in a machine-learning model (Zeng *et al.* 2024), suggesting a potential direction for future methodological improvements.

When benchmarked against RVIS, LOEUF, shet, and CoNeS, GDIv2 showed moderate correlations with all scores, reflecting partial overlap in the biological signals they capture. Among all tested metrics, GDIv2_1KGP_37 achieved the highest AUC, while CoNeS and LOEUF showed superior performance in balancing the exclusion of accessory genes and the retention of relevant genes when binarized using a threshold corresponding to the top 5% of their respective score distributions. The moderate correlation between GDIv2_1KGP_37, CoNeS and LOEUF supports their combined use. Indeed, integrating the three scores dramatically increased the exclusion of accessory genes while maintaining a very low exclusion rate for relevant genes. Further refinement by rescuing genes with LOEUF < 1 or CoNeS < 0 allowed us to retain nearly half of the initially excluded relevant genes while minimally affecting accessory gene removal. This combined strategy reduced the candidate gene space by 7.6% and the candidate variant space by up to 15% while preserving nearly all known X-linked and autosomal dominant disease genes, representing a substantial gain for rare disease analyses.

Some relevant genes were excluded by our combined strategy. Nine of these genes had an AD mode of inheritance, including *APOL1*, and *NOD2*. *APOL1* encodes the apolipoprotein L1, which is involved in resistance to infection with Trypanosoma brucei (Poli *et al.* 2025). It has been suggested that genetic variants of *APOL1* emerged as a result of positive genetic selection (Genovese *et al.* 2010, Pays *et al.* 2014, Beckerman and Susztak 2018). African-specific APOL1 gain-of-function variants protect against T b rhodesiense and T b gambiense when present in the heterozygous state, but they increase susceptibility to focal segmental glomerulosclerosis in African Americans when present in the homozygous state (Genovese *et al.* 2010). APOL1 is an example of a gene under balancing selection. By bringing an allele to an intermediate equilibrium, balancing selection may increase variability in the genomic region close to the selected locus (Hedrick 2007). *NOD2* is a central innate immune sensor involved in bacterial peptidoglycan recognition. Rare mutations in the nucleotide-binding domain of *NOD2* underlie Blau syndrome, characterized by granulomatous inflammation of the skin, joints and eyes (Miceli-Richard *et al.* 2001, Borzutzky *et al.* 2010), while common polymorphisms of *NOD2* have been associated with Crohn’s disease (Hugot *et al.* 2001, Lesage *et al.* 2002, Murillo *et al.* 2002). Some *NOD2* Crohn’s disease-risk alleles have been proposed to be maintained in the European population by balancing or recent positive selection (Jostins *et al.* 2012, Nakagome *et al.* 2012). Hence, the exclusion of *NOD2* and *APOL1* could be consistent with their evolutionary history, which can obscure constraint- and burden-based metrics.

Other limitations should be acknowledged. First, benchmarking performance depends strongly on the definition of relevant and accessory gene sets, for which no universally accepted gold standards exist, and alternative gene categorizations could influence relative performance estimates. Second, GDIv2 is currently computed at the whole-gene level. Extending GDIv2 to subgenic regions, such as protein domains, exons, or regulatory elements, may improve sensitivity for genes in which pathogenicity is driven by region-specific constraint, and align conceptually with existing region-based metrics such as subRVIS (Gussow *et al.* 2016) and the constraint coding regions (CCR) percentiles (Havrilla *et al.* 2019). Finally, GDIv2 is restricted to protein-coding genes, whereas disease-causing variants have also been identified in non–protein-coding RNAs (Nemeth *et al.* 2024, Gama-Carvalho *et al.* 2025) and other regulatory genomic elements (Chiou *et al.* 2021, Ying *et al.* 2023). Future extensions incorporating non-coding regions could broaden the applicability of the framework beyond the coding genome.

## Supplementary Material

vbag144_Supplementary_Data

## Data Availability

The data underlying this article are available at https://hgidsoft.rockefeller.edu/GDI/GDIv2.html.
