# Supplementary material for: GDIv2: improving variant selection from human exomes
Source: Bioinform Adv. 2026 May 23;6(1):vbag144. doi: 10.1093/bioadv/vbag144 (PMC13289815; doi:10.1093/bioadv/vbag144)
Supplement: vbag144_Supplementary_Data [file vbag144_supplementary_data.zip › Supplementary material.pdf]

Supplementary material

Table S1: Thresholds representing the top 5% most damaged genes for GDI, GDIv2 and other gene-level metrics (pLI, LOx10UF, CoNeS, s_{het}, RVIS)

Score	5%-cutoff threshold
GDI	>13
GDIv2_1kGP_37	>13
GDIv2_GnomAD_37	>13
GDIv2_1kGP_38	>13
GDIv2_GnomAD_38	>13
RVIS	>1.353
CoNeS	>1.559
LOx10UF	>1.813
s_{het}	<0.000828

Table S2: Descriptive information about each gene (Symbol, Gencode, Transcript selected, length of coding sequence…), GDI score, other gene-level metrics (pLI, LOx10UF, CoNeS, s_{het}, RVIS) and gene categories.

See online supplementary material

Table S3: AUC of ROC curves assessing the ability to distinguish relevant genes from accessory genes, relevant from background genes and accessory from background genes for GDI, GDIv2 scores. p-values are reported for each pairwise comparison (as implemented in pROC with bootstrap test)

**Category**	**Score1**	**Score2**	**n**	**AUC1**	**AUC2**	**P-value**
Relevant vs Accessory	GDI	GDIv2_1kGP_37	2888	0.611	0.877	$3.07x10^{-110}$
Relevant vs Accessory	GDI	GDIv2_1kGP_38	2888	0.611	0.811	$1.87x10^{-53}$
Relevant vs Accessory	GDI	GDIv2_GnomAD_37	2888	0.611	0.86	$2.77x10^{-99}$
Relevant vs Accessory	GDI	GDIv2_GnomAD_38	2888	0.611	0.82	$5.82x10^{-52}$
Relevant vs Accessory	GDIv2_1kGP_37	GDIv2_1kGP_38	2888	0.877	0.811	$8.42x10^{-19}$
Relevant vs Accessory	GDIv2_1kGP_37	GDIv2_GnomAD_37	2888	0.877	0.86	$1.64x10^{-06}$
Relevant vs Accessory	GDIv2_1kGP_37	GDIv2_GnomAD_38	2888	0.877	0.82	$2.02x10^{-12}$
Relevant vs Accessory	GDIv2_1kGP_38	GDIv2_GnomAD_37	2888	0.811	0.86	$7.11x10^{-12}$
Relevant vs Accessory	GDIv2_1kGP_38	GDIv2_GnomAD_38	2888	0.811	0.82	0.0853
Relevant vs Accessory	GDIv2_GnomAD_37	GDIv2_GnomAD_38	2888	0.86	0.82	$8.88x10^{-07}$
Relevant vs Background	GDI	GDIv2_1kGP_37	17440	0.469	0.585	$7.98x10^{-38}$
Relevant vs Background	GDI	GDIv2_1kGP_38	17440	0.469	0.579	$2.04x10^{-37}$
Relevant vs Background	GDI	GDIv2_GnomAD_37	17440	0.469	0.579	$4.24x10^{-36}$
Relevant vs Background	GDI	GDIv2_GnomAD_38	17440	0.469	0.593	$6.49x10^{-41}$
Relevant vs Background	GDIv2_1kGP_37	GDIv2_1kGP_38	17440	0.585	0.579	0.084
Relevant vs Background	GDIv2_1kGP_37	GDIv2_GnomAD_37	17440	0.585	0.579	0.00124
Relevant vs Background	GDIv2_1kGP_37	GDIv2_GnomAD_38	17440	0.585	0.593	0.0731
Relevant vs Background	GDIv2_1kGP_38	GDIv2_GnomAD_37	17440	0.579	0.579	0.838
Relevant vs Background	GDIv2_1kGP_38	GDIv2_GnomAD_38	17440	0.579	0.593	$3.66x10^{-05}$
Relevant vs Background	GDIv2_GnomAD_37	GDIv2_GnomAD_38	17440	0.579	0.593	0.0022
Accessory vs Background	GDI	GDIv2_1kGP_37	16010	0.641	0.824	$3.90x10^{-74}$
Accessory vs Background	GDI	GDIv2_1kGP_38	16010	0.641	0.758	$6.56x10^{-24}$
Accessory vs Background	GDI	GDIv2_GnomAD_37	16010	0.641	0.812	$1.41x10^{-65}$
Accessory vs Background	GDI	GDIv2_GnomAD_38	16010	0.641	0.755	$5.22x10^{-20}$
Accessory vs Background	GDIv2_1kGP_37	GDIv2_1kGP_38	16010	0.824	0.758	$5.48x10^{-18}$
Accessory vs Background	GDIv2_1kGP_37	GDIv2_GnomAD_37	16010	0.824	0.812	0.000254
Accessory vs Background	GDIv2_1kGP_37	GDIv2_GnomAD_38	16010	0.824	0.755	$8.91x10^{-17}$
Accessory vs Background	GDIv2_1kGP_38	GDIv2_GnomAD_37	16010	0.758	0.812	$7.49x10^{-13}$
Accessory vs Background	GDIv2_1kGP_38	GDIv2_GnomAD_38	16010	0.758	0.755	0.599
Accessory vs Background	GDIv2_GnomAD_37	GDIv2_GnomAD_38	16010	0.812	0.755	$9.63x10^{-12}$

Table S4: PR-AUC assessing the ability to distinguish relevant genes from accessory genes, relevant from background genes and accessory from background genes for GDI, GDIv2 scores.

**Positive class**	**Negative class**	**Comparison**	**Score**	**PR_AUC**	**Prevalence**	**x10nrichment_over_random**
Relevant	Accessory	Relevant vs Accessory	GDI	0.80	0.12	6.77
Relevant	Accessory	Relevant vs Accessory	GDIv2_1kGP_37	0.96	0.12	**8.05**
Relevant	Accessory	Relevant vs Accessory	GDIv2_1kGP_38	0.92	0.12	7.77
Relevant	Accessory	Relevant vs Accessory	GDIv2_GnomAD_37	0.95	0.12	8.00
Relevant	Accessory	Relevant vs Accessory	GDIv2_GnomAD_38	0.92	0.12	7.78
Accessory	Relevant	Accessory vs Relevant	GDI	0.32	0.04	8.10
Accessory	Relevant	Accessory vs Relevant	GDIv2_1kGP_37	0.64	0.04	**16.06**
Accessory	Relevant	Accessory vs Relevant	GDIv2_1kGP_38	0.55	0.04	13.60
Accessory	Relevant	Accessory vs Relevant	GDIv2_GnomAD_37	0.61	0.04	15.32
Accessory	Relevant	Accessory vs Relevant	GDIv2_GnomAD_38	0.59	0.04	14.66
Relevant	Background	Relevant vs Background	GDI	0.11	0.12	0.96
Relevant	Background	Relevant vs Background	GDIv2_1kGP_37	0.17	0.12	1.41
Relevant	Background	Relevant vs Background	GDIv2_1kGP_38	0.16	0.12	1.37
Relevant	Background	Relevant vs Background	GDIv2_GnomAD_37	0.16	0.12	1.39
Relevant	Background	Relevant vs Background	GDIv2_GnomAD_38	0.17	0.12	**1.43**
Accessory	Background	Accessory vs Background	GDI	0.08	0.04	1.95
Accessory	Background	Accessory vs Background	GDIv2_1kGP_37	0.15	0.04	**3.85**
Accessory	Background	Accessory vs Background	GDIv2_1kGP_38	0.12	0.04	2.98
Accessory	Background	Accessory vs Background	GDIv2_GnomAD_37	0.15	0.04	3.69
Accessory	Background	Accessory vs Background	GDIv2_GnomAD_38	0.13	0.04	3.23

Table S5: Ablation analysis: AUC of ROC curves assessing the ability of GDI (original score), GDIv1_updated (GDI recalculated on 1kGP with Vx10P annotation and canonical transcript selection), GDIv1_regressed (GDIv1_updated regressed on the length of the coding sequence), GDIv2_noregression (GDIv2_1kGP_37 without the regression on the length of the coding sequence) and GDIv2_1kGP_37 to distinguish between relevant and accessory genes. p-values are reported for each pairwise comparison (as implemented in pROC with bootstrap test)

**Comparison**	**Score 1**	**Score 2**	**AUC1**	**AUC2**	**Statistic**	**P-value**	**P-value_adjusted**
Relevant vs Accessory	GDI	GDIv1_updated	0.78	0.80	-6.08	1.19x10^{-09}	1.71x10^{-09}
Relevant vs Accessory	GDI	GDIv1_regressed	0.78	0.86	$^{-12}$.41	2.31x10^{-35}	1.33x10^{-34}
Relevant vs Accessory	GDI	GDIv2_noregression	0.78	0.79	-2.97	2.96x10^{-03}	3.29x10^{-03}
Relevant vs Accessory	GDI	GDIv2_1kGP_37	0.78	0.89	$^{-10}$.36	3.61x10^{-25}	7.22x10^{-25}
Relevant vs Accessory	GDIv1_updated	GDIv1_regressed	0.80	0.86	$^{-12}$.40	2.66x10^{-35}	1.33x10^{-34}
Relevant vs Accessory	GDIv1_updated	GDIv2_noregression	0.80	0.79	2.32	2.05x10^{-02}	2.05x10^{-02}
Relevant vs Accessory	GDIv1_updated	GDIv2_1kGP_37	0.80	0.89	-8.85	8.67x10^{-19}	1.45x10^{-18}
Relevant vs Accessory	GDIv1_regressed	GDIv2_noregression	0.86	0.79	10.87	1.55x10^{-27}	3.87x10^{-27}
Relevant vs Accessory	GDIv1_regressed	GDIv2_1kGP_37	0.86	0.89	-4.33	1.48x10^{-05}	1.85x10^{-05}
Relevant vs Accessory	GDIv2_noregression	GDIv2_1kGP_37	0.79	0.89	$^{-11}$.09	1.43x10^{-28}	4.76x10^{-28}

Table S6: Comparisons of the proportions of accessory, background or relevant genes removed by GDI and GDIv2. P-values reported were calculated using a McNemar test and adjusted for multiple testing.

Category	Score1	Score2	n	Proportion1	Proportion2	P-value	P-value_adjusted
Accessory	GDI	GDIv2_1kGP_37	729	0.13	0.25	$2.71x10^{-11}$	$2.71x10^{-10}$
Accessory	GDI	GDIv2_1kGP_38	729	0.13	0.19	$7.55x10^{-04}$	$1.51x10^{-03}$
Accessory	GDI	GDIv2_GnomAD_37	729	0.13	0.22	$1.41x10^{-08}$	$7.07x10^{-08}$
Accessory	GDI	GDIv2_GnomAD_38	729	0.13	0.22	$1.18x10^{-07}$	$3.92x10^{-07}$
Accessory	GDIv2_1kGP_37	GDIv2_1kGP_38	729	0.25	0.19	$1.14x10^{-05}$	$2.85x10^{-05}$
Accessory	GDIv2_1kGP_37	GDIv2_GnomAD_37	729	0.25	0.22	$8.72x10^{-02}$	$9.69x10^{-02}$
Accessory	GDIv2_1kGP_37	GDIv2_GnomAD_38	729	0.25	0.22	$7.80x10^{-02}$	$9.69x10^{-02}$
Accessory	GDIv2_1kGP_38	GDIv2_GnomAD_37	729	0.19	0.22	$2.68x10^{-03}$	$4.47x10^{-03}$
Accessory	GDIv2_1kGP_38	GDIv2_GnomAD_38	729	0.19	0.22	$5.52x10^{-02}$	$7.89x10^{-02}$
Accessory	GDIv2_GnomAD_37	GDIv2_GnomAD_38	729	0.22	0.22	$5.97x10^{-01}$	$5.97x10^{-01}$
Background	GDI	GDIv2_1kGP_37	15281	0.04	0.04	$8.78x10^{-01}$	$9.50x10^{-01}$
Background	GDI	GDIv2_1kGP_38	15281	0.04	0.04	$9.50x10^{-01}$	$9.50x10^{-01}$
Background	GDI	GDIv2_GnomAD_37	15281	0.04	0.04	$7.34x10^{-01}$	$9.50x10^{-01}$
Background	GDI	GDIv2_GnomAD_38	15281	0.04	0.04	$7.22x10^{-01}$	$9.50x10^{-01}$
Background	GDIv2_1kGP_37	GDIv2_1kGP_38	15281	0.04	0.04	$9.23x10^{-01}$	$9.50x10^{-01}$
Background	GDIv2_1kGP_37	GDIv2_GnomAD_37	15281	0.04	0.04	$7.87x10^{-01}$	$9.50x10^{-01}$
Background	GDIv2_1kGP_37	GDIv2_GnomAD_38	15281	0.04	0.04	$5.12x10^{-01}$	$9.50x10^{-01}$
Background	GDIv2_1kGP_38	GDIv2_GnomAD_37	15281	0.04	0.04	$7.22x10^{-01}$	$9.50x10^{-01}$
Background	GDIv2_1kGP_38	GDIv2_GnomAD_38	15281	0.04	0.04	$5.74x10^{-01}$	$9.50x10^{-01}$
Background	GDIv2_GnomAD_37	GDIv2_GnomAD_38	15281	0.04	0.04	$3.81x10^{-01}$	$9.50x10^{-01}$
Relevant	GDI	GDIv2_1kGP_37	2159	0.07	0.03	$1.02x10^{-07}$	$5.12x10^{-07}$
Relevant	GDI	GDIv2_1kGP_38	2159	0.07	0.04	$1.51x10^{-06}$	$3.76x10^{-06}$
Relevant	GDI	GDIv2_GnomAD_37	2159	0.07	0.04	$2.77x10^{-07}$	$9.22x10^{-07}$
Relevant	GDI	GDIv2_GnomAD_38	2159	0.07	0.03	$4.92x10^{-12}$	$4.92x10^{-11}$
Relevant	GDIv2_1kGP_37	GDIv2_1kGP_38	2159	0.03	0.04	$3.41x10^{-01}$	$4.26x10^{-01}$
Relevant	GDIv2_1kGP_37	GDIv2_GnomAD_37	2159	0.03	0.04	$5.51x10^{-01}$	$6.12x10^{-01}$
Relevant	GDIv2_1kGP_37	GDIv2_GnomAD_38	2159	0.03	0.03	$4.72x10^{-02}$	$6.74x10^{-02}$

Relevant	GDIv2_1kGP_38	GDIv2_GnomAD_37	2159	0.04	0.04	$7.79x10^{-01}$	$7.79x10^{-01}$
Relevant	GDIv2_1kGP_38	GDIv2_GnomAD_38	2159	0.04	0.03	$1.21x10^{-03}$	$2.42x10^{-03}$
Relevant	GDIv2_GnomAD_37	GDIv2_GnomAD_38	2159	0.04	0.03	$7.65x10^{-03}$	$1.28x10^{-02}$

Table S7: Median and interquartile range (IQR) for LOx10UF, CoNeS, s_{het}, and RVIS across different gene categories and mode of inheritance for relevant genes

	**Accessory**		**Background**		**Relevant**		**AR**		**AD**		**XL**	
**score**	**n**	**Median (IQR)**	**n**	**Median (IQR)**	**n**	**Median (IQR)**	**n**	**Median (IQR)**	**n**	**Median (IQR)**	**n**	**Median (IQR)**
GDIv2_1kGP_37	328	7.8 (6.4)	14042	2.8 (4.3)	2104	2.0 (3.5)	1250	2.8 (3.9)	630	1.1 (2.3)	137	1.2 (2.8)
GDIv2_GnomAD_38	328	6.5 (6.3)	14042	2.7 (4.2)	2104	1.9 (3.5)	1250	2.5 (4)	630	1 (2.3)	137	1.2 (3)
LOx10UF_Gnomadv4.1.1	328	1.3 (0.7)	14042	0.9 (0.6)	2104	0.8 (0.6)	1250	0.9 (0.3)	630	0.4 (0.4)	137	0.3 (0.2)
s_{het}	328	0.003 (0.004)	14042	0.01 (0.05)	2104	0.02 (0.004)	1250	0.006 (0.02)	630	0.1 (0.2)	137	0.2 (0.1)
RVIS	328	0.5 (0.7)	14042	-0.03 (0.8)	2104	-0.2 (0.9)	1250	-0.01 (0.8)	630	-0.7 (1.1)	137	-0.4 (1.1)
CoNeS	328	1.0 (1.1)	14042	0.008 (1.4)	2104	-0.3 (1.5)	1250	0.1 (0.9)	630	-1.4 (1.1)	137	-1.4 (0.6)

Table S8: AUC of ROC curves assessing the ability to distinguish relevant genes from accessory genes, relevant from background genes and accessory from background genes for GDIv2_1kGP_37, GDIv2_GnomAD_38, LOx10UF, CoNeS, s_{het}, and RVIS. p-values are reported for each pairwise comparison (as implemented in pROC with bootstrap test).

**Comparison**	**Score1**	**Score2**	**n**	**AUC1**	**AUC2**	**P_value**
Relevant vs Accessory	GDIv2_1kGP_37	GDIv2_GnomAD_38	2432	0.86	0.82	$4.86x10^{-05}$
Relevant vs Accessory	GDIv2_1kGP_37	RVIS	2432	0.86	0.80	$2.68x10^{-07}$
Relevant vs Accessory	GDIv2_1kGP_37	LOx10UF	2432	0.86	0.85	0.456
Relevant vs Accessory	GDIv2_1kGP_37	CoNeS	2432	0.86	0.85	0.466
Relevant vs Accessory	GDIv2_1kGP_37	s_{het}	2432	0.86	0.79	$8.26x10^{-10}$
Relevant vs Accessory	GDIv2_GnomAD_38	RVIS	2432	0.82	0.80	0.444
Relevant vs Accessory	GDIv2_GnomAD_38	LOx10UF	2432	0.82	0.85	0.0174
Relevant vs Accessory	GDIv2_GnomAD_38	CoNeS	2432	0.82	0.85	0.0112
Relevant vs Accessory	GDIv2_GnomAD_38	s_{het}	2432	0.82	0.79	0.0525
Relevant vs Accessory	RVIS	LOx10UF	2432	0.80	0.85	0.000564
Relevant vs Accessory	RVIS	CoNeS	2432	0.80	0.85	$3.91x10^{-08}$
Relevant vs Accessory	RVIS	shet	2432	0.80	0.79	0.152
Relevant vs Accessory	LOx10UF	CoNeS	2432	0.85	0.85	0.917
Relevant vs Accessory	LOx10UF	s_{het}	2432	0.85	0.79	$1.45x10^{-07}$
Relevant vs Accessory	CoNeS	s_{het}	2432	0.85	0.79	$3.68x10^{-10}$
Relevant vs Background	GDIv2_1kGP_37	GDIv2_GnomAD_38	16146	0.58	0.58	0.0795
Relevant vs Background	GDIv2_1kGP_37	RVIS	16146	0.58	0.58	0.404
Relevant vs Background	GDIv2_1kGP_37	LOx10UF	16146	0.58	0.61	$6.10x10^{-07}$
Relevant vs Background	GDIv2_1kGP_37	CoNeS	16146	0.58	0.59	0.0126
Relevant vs Background	GDIv2_1kGP_37	s_{het}	16146	0.58	0.58	0.225
Relevant vs Background	GDIv2_GnomAD_38	RVIS	16146	0.58	0.58	0.849
Relevant vs Background	GDIv2_GnomAD_38	LOx10UF	16146	0.58	0.61	$9.73x10^{-05}$
Relevant vs Background	GDIv2_GnomAD_38	CoNeS	16146	0.58	0.59	0.161
Relevant vs Background	GDIv2_GnomAD_38	s_{het}	16146	0.58	0.58	0.879
Relevant vs Background	RVIS	LOx10UF	16146	0.58	0.61	$2.80x10^{-05}$
Relevant vs Background	RVIS	CoNeS	16146	0.58	0.59	0.0252

Relevant vs Background	RVIS	s_{het}	16146	0.58	0.58	0.689
Relevant vs Background	LOx10UF	CoNeS	16146	0.61	0.59	0.000779
Relevant vs Background	LOx10UF	s_{het}	16146	0.61	0.58	$7.74x10^{-13}$
Relevant vs Background	CoNeS	s_{het}	16146	0.59	0.58	0.0154
Accessory vs Background	GDIv2_1kGP_37	GDIv2_GnomAD_38	14370	0.81	0.75	$1.56x10^{-06}$
Accessory vs Background	GDIv2_1kGP_37	RVIS	14370	0.81	0.76	$4.45x10^{-06}$
Accessory vs Background	GDIv2_1kGP_37	LOx10UF	14370	0.81	0.77	0.00221
Accessory vs Background	GDIv2_1kGP_37	CoNeS	14370	0.81	0.79	0.0849
Accessory vs Background	GDIv2_1kGP_37	s_{het}	14370	0.81	0.69	$2.99x10^{-22}$
Accessory vs Background	GDIv2_GnomAD_38	RVIS	14370	0.75	0.76	0.72
Accessory vs Background	GDIv2_GnomAD_38	LOx10UF	14370	0.75	0.77	0.303
Accessory vs Background	GDIv2_GnomAD_38	CoNeS	14370	0.75	0.79	0.0174
Accessory vs Background	GDIv2_GnomAD_38	s_{het}	14370	0.75	0.69	$5.27x10^{-05}$
Accessory vs Background	RVIS	LOx10UF	14370	0.76	0.77	0.441
Accessory vs Background	RVIS	CoNeS	14370	0.76	0.79	0.00036
Accessory vs Background	RVIS	s_{het}	14370	0.76	0.69	$2.34x10^{-07}$
Accessory vs Background	LOx10UF	CoNeS	14370	0.77	0.79	0.122
Accessory vs Background	LOx10UF	s_{het}	14370	0.77	0.69	$4.17x10^{-08}$
Accessory vs Background	CoNeS	s_{het}	14370	0.79	0.69	$9.43x10^{-18}$

Table S9: PR-AUC assessing the ability to distinguish relevant genes from accessory genes, relevant from background genes and accessory from background genes for GDIv2_1kGP_37, GDIv2_GnomAD_38, LOx10UF, CoNeS, s_{het}, and RVIS.

**Positive class**	**Negative class**	**Comparison**	**Score**	**PR_AUC**	**Prevalence**	**x10nrichment_over_random**
Relevant	Accessory	Relevant vs Accessory	GDIv2_1kGP_37	0.98	0.13	7.64
Relevant	Accessory	Relevant vs Accessory	GDIv2_GnomAD_38	0.96	0.13	7.54
Relevant	Accessory	Relevant vs Accessory	RVIS	0.96	0.13	7.55
Relevant	Accessory	Relevant vs Accessory	LOx10UF	0.97	0.13	7.58
Relevant	Accessory	Relevant vs Accessory	CoNeS	0.97	0.13	7.61
Relevant	Accessory	Relevant vs Accessory	s_{het}	0.96	0.13	7.52
Accessory	Relevant	Accessory vs Relevant	GDIv2_1kGP_37	0.43	0.02	21.53
Accessory	Relevant	Accessory vs Relevant	GDIv2_GnomAD_38	0.38	0.02	19.10
Accessory	Relevant	Accessory vs Relevant	RVIS	0.30	0.02	15.05
Accessory	Relevant	Accessory vs Relevant	LOx10UF	0.56	0.02	28.13
Accessory	Relevant	Accessory vs Relevant	CoNeS	0.50	0.02	25.36
Accessory	Relevant	Accessory vs Relevant	s_{het}	0.30	0.02	15.08
Relevant	Background	Relevant vs Background	GDIv2_1kGP_37	0.17	0.13	1.34
Relevant	Background	Relevant vs Background	GDIv2_GnomAD_38	0.17	0.13	1.36
Relevant	Background	Relevant vs Background	RVIS	0.19	0.13	1.52
Relevant	Background	Relevant vs Background	LOx10UF	0.22	0.13	1.69
Relevant	Background	Relevant vs Background	CoNeS	0.21	0.13	1.66
Relevant	Background	Relevant vs Background	s_{het}	0.18	0.13	1.44
Accessory	Background	Accessory vs Background	GDIv2_1kGP_37	0.08	0.02	3.80
Accessory	Background	Accessory vs Background	GDIv2_GnomAD_38	0.06	0.02	3.09
Accessory	Background	Accessory vs Background	RVIS	0.05	0.02	2.66
Accessory	Background	Accessory vs Background	LOx10UF	0.09	0.02	4.30
Accessory	Background	Accessory vs Background	CoNeS	0.09	0.02	4.44
Accessory	Background	Accessory vs Background	s_{het}	0.04	0.02	1.85

Table S10: Comparisons of the proportions of accessory, background or relevant genes (all or stratified by mode of inheritance) removed by GDIv2_1kGP_37, GDIv2_GnomAD_38, LOx10UF, CoNeS, s_{het}, and RVIS. P-values reported were calculated using a McNemar test and adjusted for multiple testing

**Category**	**Score1**	**Score2**	**n**	**Proportion1**	**Proportion2**	**P-value**	**P-value_adjusted**
Accessory	GDIv2_1kGP_37	GDIv2_GnomAD_38	328	0.19	0.13	$1.01x10^{-02}$	$1.90x10^{-02}$
Accessory	GDIv2_1kGP_37	RVIS	328	0.19	0.15	$8.72x10^{-02}$	$1.09x10^{-01}$
Accessory	GDIv2_1kGP_37	LOx10UFv4.1	328	0.19	0.23	$3.47x10^{-01}$	$4.00x10^{-01}$
Accessory	GDIv2_1kGP_37	CoNeS	328	0.19	0.26	$2.09x10^{-02}$	$2.85x10^{-02}$
Accessory	GDIv2_1kGP_37	s_{het}	328	0.19	0.07	$8.68x10^{-06}$	$2.61x10^{-05}$
Accessory	GDIv2_GnomAD_38	RVIS	328	0.13	0.15	$6.94x10^{-01}$	$6.94x10^{-01}$
Accessory	GDIv2_GnomAD_38	LOx10UFv4.1	328	0.13	0.23	$2.50x10^{-03}$	$5.36x10^{-03}$
Accessory	GDIv2_GnomAD_38	CoNeS	328	0.13	0.26	$1.58x10^{-06}$	$7.91x10^{-06}$
Accessory	GDIv2_GnomAD_38	s_{het}	328	0.13	0.07	$1.42x10^{-02}$	$2.13x10^{-02}$
Accessory	RVIS	LOx10UFv4.1	328	0.15	0.23	$1.33x10^{-02}$	$2.13x10^{-02}$
Accessory	RVIS	CoNeS	328	0.15	0.26	$2.91x10^{-06}$	$1.09x10^{-05}$
Accessory	RVIS	s_{het}	328	0.15	0.07	$2.12x10^{-03}$	$5.29x10^{-03}$
Accessory	LOx10UFv4.1	CoNeS	328	0.23	0.26	$3.82x10^{-01}$	$4.09x10^{-01}$
Accessory	LOx10UFv4.1	s_{het}	328	0.23	0.07	$5.70x10^{-07}$	$4.28x10^{-06}$
Accessory	CoNeS	s_{het}	328	0.26	0.07	$1.22x10^{-10}$	$1.82x10^{-09}$
Background	GDIv2_1kGP_37	GDIv2_GnomAD_38	14042	0.04	0.04	$4.95x10^{-01}$	$5.20x10^{-01}$
Background	GDIv2_1kGP_37	RVIS	14042	0.04	0.05	$2.65x10^{-03}$	$4.96x10^{-03}$
Background	GDIv2_1kGP_37	LOx10UFv4.1	14042	0.04	0.04	$2.41x10^{-01}$	$3.01x10^{-01}$
Background	GDIv2_1kGP_37	CoNeS	14042	0.04	0.04	$2.61x10^{-01}$	$3.02x10^{-01}$
Background	GDIv2_1kGP_37	s_{het}	14042	0.04	0.06	$5.27x10^{-13}$	$2.63x10^{-12}$
Background	GDIv2_GnomAD_38	RVIS	14042	0.04	0.05	$3.16x10^{-04}$	$6.78x10^{-04}$
Background	GDIv2_GnomAD_38	LOx10UFv4.1	14042	0.04	0.04	$5.20x10^{-01}$	$5.20x10^{-01}$
Background	GDIv2_GnomAD_38	CoNeS	14042	0.04	0.04	$7.62x10^{-02}$	$1.04x10^{-01}$
Background	GDIv2_GnomAD_38	s_{het}	14042	0.04	0.06	$6.19x10^{-15}$	$4.64x10^{-14}$
Background	RVIS	LOx10UFv4.1	14042	0.05	0.04	$1.63x10^{-04}$	$4.08x10^{-04}$

Background	RVIS	CoNeS	14042	0.05	0.04	$2.35x10^{-02}$	$3.91x10^{-02}$
Background	RVIS	s_{het}	14042	0.05	0.06	$1.42x10^{-06}$	$4.26x10^{-06}$
Background	LOx10UFv4.1	CoNeS	14042	0.04	0.04	$2.70x10^{-02}$	$4.06x10^{-02}$
Background	LOx10UFv4.1	s_{het}	14042	0.04	0.06	8.05x10-16	$1.21x10^{-14}$
Background	CoNeS	s_{het}	14042	0.04	0.06	$3.86x10^{-11}$	$1.45x10^{-10}$
Relevant	GDIv2_1kGP_37	GDIv2_GnomAD_38	2104	0.03	0.02	$5.68x10^{-02}$	$7.74x10^{-02}$
Relevant	GDIv2_1kGP_37	RVIS	2104	0.03	0.05	$3.20x10^{-03}$	$5.33x10^{-03}$
Relevant	GDIv2_1kGP_37	LOx10UFv4.1	2104	0.03	0.01	$1.01x10^{-06}$	$5.07x10^{-06}$
Relevant	GDIv2_1kGP_37	CoNeS	2104	0.03	0.01	$2.20x10^{-04}$	$5.50x10^{-04}$
Relevant	GDIv2_1kGP_37	s_{het}	2104	0.03	0.02	$7.63x10^{-02}$	$9.52x10^{-02}$
Relevant	GDIv2_GnomAD_38	RVIS	2104	0.02	0.05	$4.57x10^{-06}$	$1.37x10^{-05}$
Relevant	GDIv2_GnomAD_38	LOx10UFv4.1	2104	0.02	0.01	$4.19x10^{-04}$	$8.98x10^{-04}$
Relevant	GDIv2_GnomAD_38	CoNeS	2104	0.02	0.01	$4.05x10^{-02}$	$6.08x10^{-02}$
Relevant	GDIv2_GnomAD_38	s_{het}	2104	0.02	0.02	$9.11x10^{-01}$	$9.11x10^{-01}$
Relevant	RVIS	LOx10UFv4.1	2104	0.05	0.01	$4.98x10^{-14}$	$7.48x10^{-13}$
Relevant	RVIS	CoNeS	2104	0.05	0.01	$1.37x10^{-13}$	$1.03x10^{-12}$
Relevant	RVIS	s_{het}	2104	0.05	0.02	$3.30x10^{-06}$	$1.24x10^{-05}$
Relevant	LOx10UFv4.1	CoNeS	2104	0.01	0.01	$9.73x10^{-02}$	$1.04x10^{-01}$
Relevant	LOx10UFv4.1	s_{het}	2104	0.01	0.02	$7.12x10^{-04}$	$1.34x10^{-03}$
Relevant	CoNeS	s_{het}	2104	0.01	0.02	$8.25x10^{-02}$	$9.52x10^{-02}$
AR	GDIv2_1kGP_37	GDIv2_GnomAD_38	1250	0.04	0.03	$6.06x10^{-02}$	$7.58x10^{-02}$
AR	GDIv2_1kGP_37	RVIS	1250	0.04	0.06	$1.52x10^{-02}$	$2.53x10^{-02}$
AR	GDIv2_1kGP_37	LOx10UFv4.1	1250	0.04	0.01	$4.67x10^{-07}$	$2.33x10^{-06}$
AR	GDIv2_1kGP_37	CoNeS	1250	0.04	0.02	$6.06x10^{-04}$	$1.30x10^{-03}$
AR	GDIv2_1kGP_37	s_{het}	1250	0.04	0.03	$4.22x10^{-02}$	$5.75x10^{-02}$
AR	GDIv2_GnomAD_38	RVIS	1250	0.03	0.06	$6.88x10^{-05}$	$2.06x10^{-04}$
AR	GDIv2_GnomAD_38	LOx10UFv4.1	1250	0.03	0.01	$1.49x10^{-04}$	$3.73x10^{-04}$
AR	GDIv2_GnomAD_38	CoNeS	1250	0.03	0.02	$6.60x10^{-02}$	$7.61x10^{-02}$

AR	GDIv2_GnomAD_38	s_{het}	1250	0.03	0.03	$6.99x10^{-01}$	$6.99x10^{-01}$
AR	RVIS	LOx10UFv4.1	1250	0.06	0.01	$8.14x10^{-13}$	$1.22x10^{-11}$
AR	RVIS	CoNeS	1250	0.06	0.02	$3.47x10^{-11}$	$2.60x10^{-10}$
AR	RVIS	s_{het}	1250	0.06	0.03	$1.71x10^{-05}$	$6.40x10^{-05}$
AR	LOx10UFv4.1	CoNeS	1250	0.01	0.02	$3.11x10^{-02}$	$4.67x10^{-02}$
AR	LOx10UFv4.1	s_{het}	1250	0.01	0.03	$7.94x10^{-04}$	$1.49x10^{-03}$
AR	CoNeS	s_{het}	1250	0.02	0.03	$2.03x10^{-01}$	$2.18x10^{-01}$
AD	GDIv2_1kGP_37	GDIv2_GnomAD_38	630	0.01	0.01	1	1
AD	GDIv2_1kGP_37	RVIS	630	0.01	0.02	$2.67x10^{-01}$	1
AD	GDIv2_1kGP_37	LOx10UFv4.1	630	0.01	0.01	1	1
AD	GDIv2_1kGP_37	CoNeS	630	0.01	0.01	$6.83x10^{-01}$	1
AD	GDIv2_1kGP_37	s_{het}	630	0.01	0.01	1	1
AD	GDIv2_GnomAD_38	RVIS	630	0.01	0.02	$3.02x10^{-01}$	1
AD	GDIv2_GnomAD_38	LOx10UFv4.1	630	0.01	0.01	1	1
AD	GDIv2_GnomAD_38	CoNeS	630	0.01	0.01	$7.24x10^{-01}$	1
AD	GDIv2_GnomAD_38	s_{het}	630	0.01	0.01	1	1
AD	RVIS	LOx10UFv4.1	630	0.02	0.01	$4.53x10^{-01}$	1
AD	RVIS	CoNeS	630	0.02	0.01	$7.04x10^{-02}$	1
AD	RVIS	s_{het}	630	0.02	0.01	$2.67x10^{-01}$	1
AD	LOx10UFv4.1	CoNeS	630	0.01	0.01	$5.05x10^{-01}$	1
AD	LOx10UFv4.1	s_{het}	630	0.01	0.01	1	1
AD	CoNeS	s_{het}	630	0.01	0.01	$7.24x10^{-01}$	1
XL	GDIv2_1kGP_37	GDIv2_GnomAD_38	137	0.01	0.01	1	1
XL	GDIv2_1kGP_37	RVIS	137	0.01	0.03	$3.71x10^{-01}$	$8.91x10^{-01}$
XL	GDIv2_1kGP_37	LOx10UFv4.1	137	0.01	0.00	1	1
XL	GDIv2_1kGP_37	CoNeS	137	0.01	0.00	1	1
XL	GDIv2_1kGP_37	s_{het}	137	0.01	0.00	1	1
XL	GDIv2_GnomAD_38	RVIS	137	0.01	0.03	$2.48x10^{-01}$	$7.45x10^{-01}$

XL	GDIv2_GnomAD_38	LOx10UFv4.1	137	0.01	0.00	1	1
XL	GDIv2_GnomAD_38	CoNeS	137	0.01	0.00	1	1
XL	GDIv2_GnomAD_38	s_{het}	137	0.01	0.00	1	1
XL	RVIS	LOx10UFv4.1	137	0.03	0.00	1.34×10^{-01}	5.34×10^{-01}
XL	RVIS	CoNeS	137	0.03	0.00	1.34×10^{-01}	5.34×10^{-01}
XL	RVIS	s_{het}	137	0.03	0.00	1.34×10^{-01}	5.34×10^{-01}
XL	LOx10UFv4.1	CoNeS	137	0.00	0.00	NA	NA
XL	LOx10UFv4.1	s_{het}	137	0.00	0.00	NA	NA
XL	CoNeS	s_{het}	137	0.00	0.00	NA	NA

Table S11: Detailed results for each fold of the 5-Fold cross-validation performance assessment of the filtering and rescue strategy based on GDIv2_1kGP_37, LOx10UF, and CoNeS

Fold	N genes	N accessory genes	N relevant genes	N accessory genes excluded	N relevant genes excluded	% accessory genes excluded	% relevant genes excluded
1	487	66	421	17	9	25.8%	2.1%
2	487	66	421	28	9	42.4%	2.1%
3	487	66	421	38	8	57.6%	1.9%
4	486	65	421	26	10	40.0%	2.4%
5	485	65	420	31	15	47.7%	3.6%
Total	486.4	65.6	420.8	28	10.2	42.7%	2.4%

Table S12: Summary of the number of allele counts excluded from the 1kGP data aligned on GRCh37 and GRCh38 when removing genes excluded by the final filtering and rescue strategy based on GDIv2_1kGP_37, LOx10UF, and CoNeS.

Genome version	Frequency filter	Sum of allele counts	Sum of allele counts in excluded genes	% excluded
GRCh38	none	56498404	8589427	15.2
GRCh38	0.05	6135737	679657	11.1
GRCh38	0.01	2897243	273897	9.5
GRCh38	0.001	1045321	80981	7.7
GRCh37	none	89917455	11772581	13.1
GRCh37	0.05	4167535	488460	11.7
GRCh37	0.01	1975277	197294	10
GRCh37	0.001	728493	59674	8.2

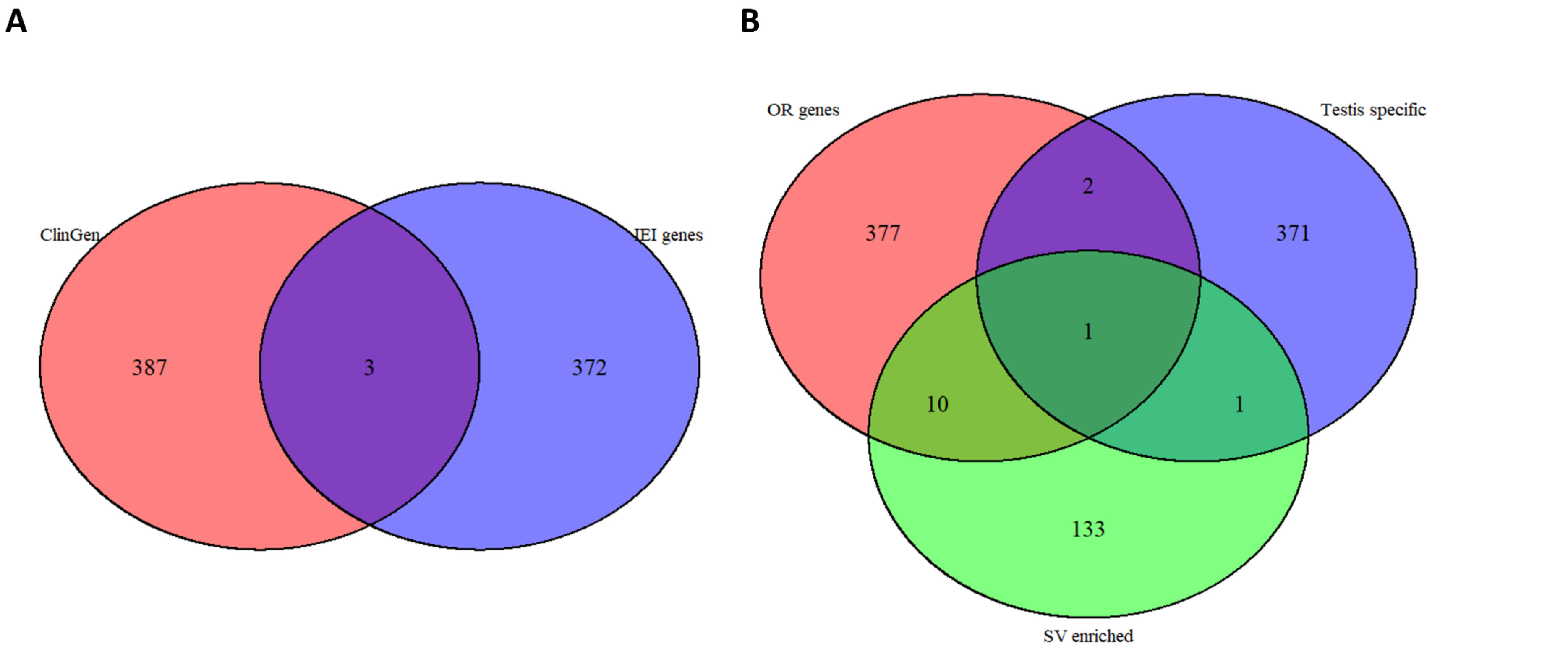

Figure S1: Definition of human gene categories. Relevant genes (A) are defined as the union of genes curated in the Gene-Disease Validity classifications (accessed on 2026/04/06) from the Clinical Genome Resource (www.clinicalgenome.org) (Rehm, et al., 2015) with a Definitive or Strong level of evidence, and a set of well-established inborn errors of immunity genes (Poli, et al., 2025). Accessory genes (B) are defined as the union of: 1) Olfactory receptor (OR) genes; 2) Testis-specific protein-coding genes; and 3) Genes frequently deleted in the general population (Structural variants [SV] enriched)

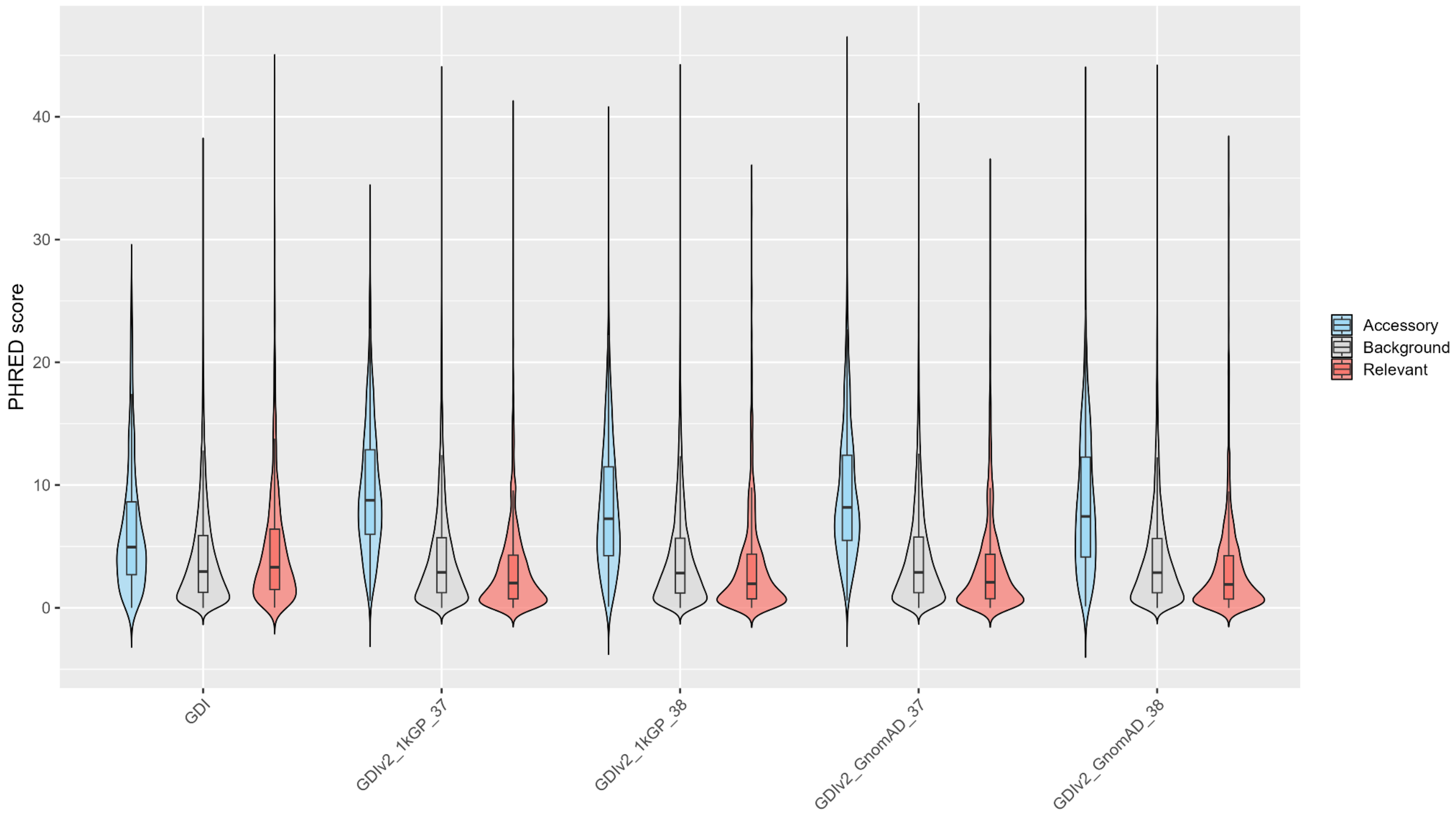

Figure S2: Distribution of GDI and GDIv2 according to gene category (background, relevant and accessory)

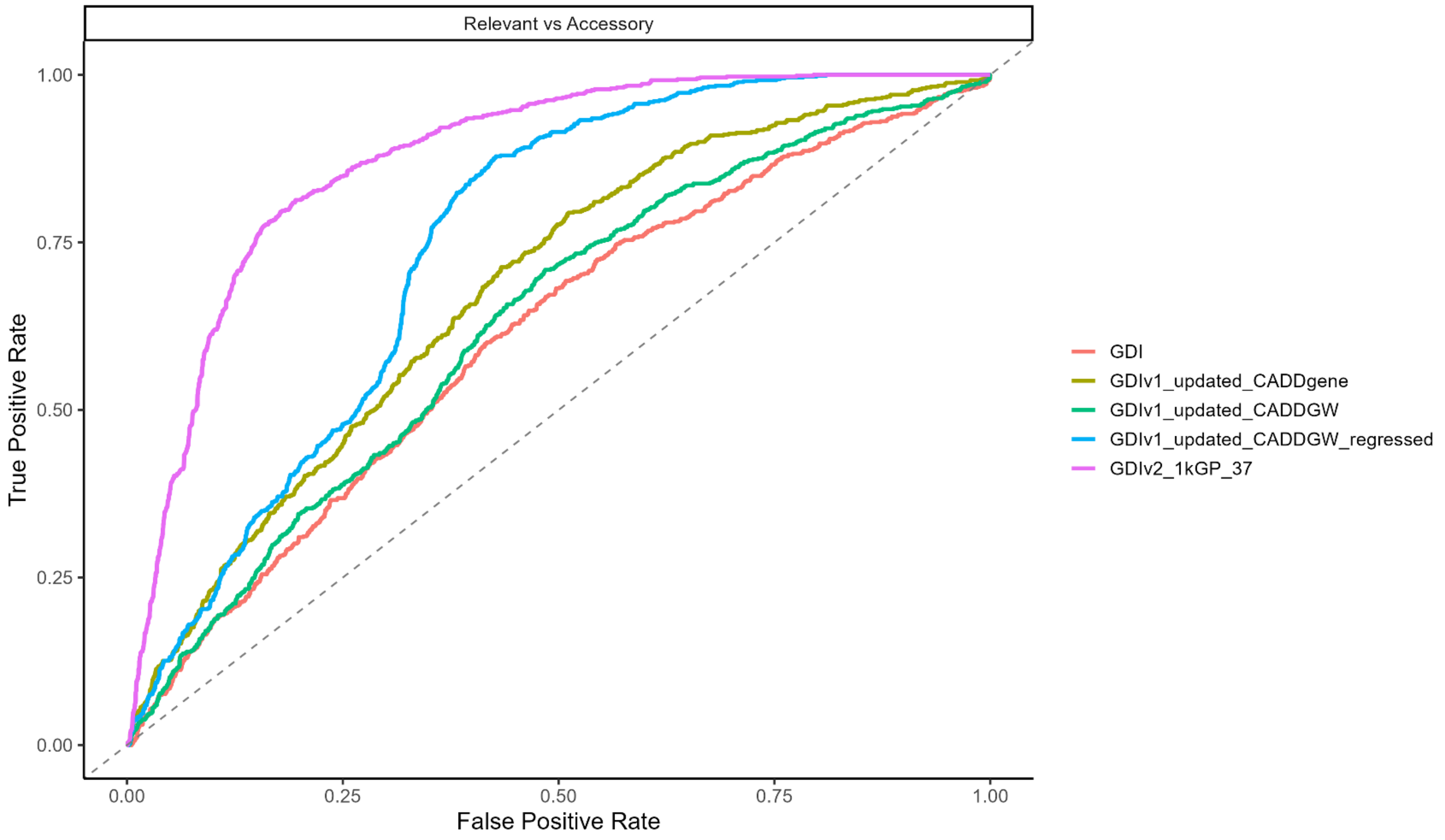

Figure S3: Ablation analysis: ROC curves assessing the ability of GDI (original score), GDIv1_updated_CADDGW (GDI recalculated on 1kGP with Vx10P annotation and canonical transcript selection), GDIv1_updated_CADDgene (GDI recalculated on 1kGP with Vx10P annotation and canonical transcript selection and gene-level CADD normalization), GDIv1_updated_CADDGW (GDIv1_updated regressed on the length of the transcript), and GDIv2_1kGP_37 to distinguish between relevant and accessory genes

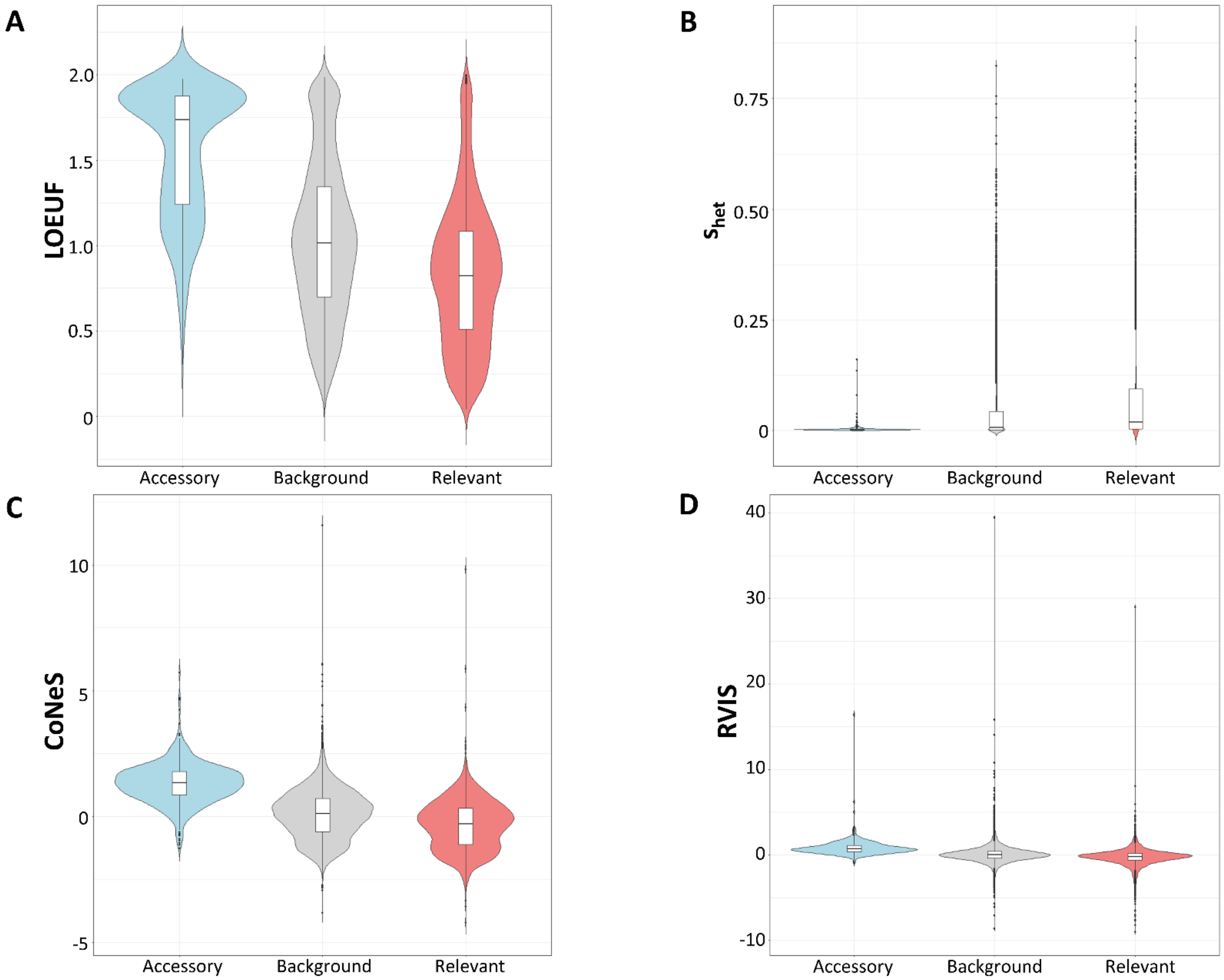

Figure S4: Distribution of LOx10UF (A), s_{het} (B), CoNeS (C) and RVIS (D) according to gene category (background, relevant and accessory)
